# Optimizing fluorescent protein expression for quantitative fluorescence microscopy and spectroscopy using herpes simplex thymidine kinase promoter sequences

**DOI:** 10.1002/2211-5463.12432

**Published:** 2018-05-08

**Authors:** Rizwan Ali, Sivaramakrishnan Ramadurai, Frank Barry, Heinz Peter Nasheuer

**Affiliations:** ^1^ Systems Biology Ireland NUI Galway Ireland; ^2^ Biochemistry School of Natural Sciences and Centre for Chromosome Biology National University of Ireland Galway Ireland; ^3^ Regenerative Medicine Institute National University of Ireland Galway Ireland; ^4^ Present address: Medical Core Facility & Research Platforms King Abdullah International Medical Research Center National Guard Health Affairs P.O. Box 3660 Riyadh 11481 Mail Code 1515 Saudi Arabia; ^5^ Present address: School of Chemical Sciences Dublin City University Dublin‐9 Ireland

**Keywords:** Fluorescence (cross‐)correlation spectroscopy, green fluorescent protein, mCherry, promoter sequences, quantitative fluorescence microscopy

## Abstract

The modulation of expression levels of fluorescent fusion proteins (FFPs) is central for recombinant DNA technologies in modern biology as overexpression of proteins contributes to artifacts in biological experiments. In addition, some microscopy techniques such as fluorescence correlation spectroscopy (FCS) and single‐molecule‐based techniques are very sensitive to high expression levels of FFPs. To reduce the levels of recombinant protein expression in comparison with the commonly used, very strong CMV promoter, the herpes simplex virus thymidine kinase (TK) gene promoter, and mutants thereof were analyzed. Deletion mutants of the TK promoter were constructed and introduced into the Gateway^®^ system for ectopic expression of enhanced green fluorescent protein (eGFP), monomeric cherry (mCherry), and FFPs containing these FPs. Two promoter constructs, TK2ST and TKTSC, were established, which have optimal low expression levels suitable for FCS studies in U2OS, HeLa CCL2, NIH 3T3, and BALB/c cells. Interestingly, when tested in these four cell lines, promoter constructs having a deletion within TK gene 5′‐UTR showed significantly higher protein expression levels than the equivalent constructs lacking this deletion. This suggests that a negative regulatory element is localized within the TK gene 5′‐UTR.

**Abbreviations**
AhR: aryl hydrocarbon receptor
CMV: cytomegalovirus immediate‐early
eGFP: enhanced green fluorescent protein
FCCS: fluorescence cross‐correlation spectroscopy
FCS: fluorescence correlation spectroscopy
FFP: fluorescent fusion protein
Fl: confocal fluorescence microscopy detection
FP: fluorescent protein
GW: Gateway®
Hif‐1: hypoxia‐inducible factor 1
HSV: herpes simplex virus
LS: linker scanning
mCherry: monomeric cherry
MSCs: mouse stem cells
PMT: photomultiplier tube
SV40: simian virus 40
TK: herpes simplex virus thymidine kinase
VDR/RXR: vitamin D receptor/retinoid X receptor

The discovery of fluorescent proteins (FPs) and their development into tools for modern imaging technologies have revolutionized modern biology and life sciences, and their use has significantly enhanced the understanding of a variety of cellular processes 1, 2. Green fluorescent protein (GFP), the first studied FP, was derived from the jellyfish *Aequorea victoria* and was later exhaustively mutated to improve its fluorescence parameters yielding the so‐called enhanced GFP (eGFP) and a variety of other fluorescent proteins differing in color 3. In recent years, the biophysical properties of eGFP, including hydrodynamic and fluorescence properties, have been studied in detail 1, 2, 4. eGFP and mCherry derived from DsRed have been the most widely used FPs for live‐cell imaging and the analysis and quantification of cellular processes 3. These two FPs and derivatives thereof are nontoxic, monomeric, and biochemically inert; they do not interact with most cellular processes and are therefore desirable for live‐cell assays 2. eGFP and mCherry have been fused to numerous cellular proteins to form fluorescent fusion proteins (FFPs) in cells and animals, to study the functions of a given protein and to act as marker proteins on cellular and animal‐wide levels 5, 6, 7, 8.

Fluorescence correlation spectroscopy (FCS) is a single‐molecule technique widely used to study mobility dynamics of proteins and biomolecules in the living cells 9, 10, 11, 12, and dual‐color fluorescence cross‐correlation spectroscopy (FCCS) has been used to measure protein–protein interactions in solution, membranes, and living cells 13, 14. In these quantitative microscopy techniques, recombinant fusion fluorescent protein expression levels are critical as elevated expression levels of fluorescent proteins diminish their suitability to perform FCS. Moreover, high expression levels may contribute, in addition to technical limitations, to concentration‐dependent artifacts in cell biological investigations and visualization 1, 5, 10, 11, 13, 15. The calculated diffusion coefficient determined by FCS describes aggregation and oligomeric state of protein 16, multiprotein complex formation 10, 17, 18, hindered diffusion, and protein–protein interaction in various solvents and cellular environments 19, 20, 21, 22. Importantly, low expression levels of FFPs are required for single‐molecule applications such as FCS and FCCS 23, 24. The basic requirement to be able to perform FCS is that the number of observed fluorescent molecules is low enough that each of them contributes substantially to the measured signal 14, 25. Only under this condition, analyses of spontaneous and noncoordinated fluctuations can be performed, which is the basis for FCS. Therefore, it is essential for FCS analyses that the concentrations and observation volumes are so far reduced that only few molecules are simultaneously detected in the observation volume, whereas at the same time the fluorescence photon yield per single molecule must be high enough for detection 14, 25, 26.

In confocal microscope‐based FCS, observation volume is defined by confocal optics, which is in the range of 0.25–1 femtoliter (fl) and ideal for subcellular‐level information, and which limits its working concentrations in the range of 5–100 nm, typical endogenous concentrations of proteins 21, 25, 27. However, by expanding the capacity of the detector system and altering mathematical corrections used, FCS measurements can be performed for concentrations even in the micromolar range 28. Commonly used cytomegalovirus immediate‐early (CMV) and simian virus 40 (SV40) promoters lead to overexpression of proteins, which might be suitable for imaging studies but is not ideal for FCS 5, 9, 15, 27. Importantly, overexpression of proteins is not required for FCS systems and interferes negatively with FCS measurements 6. In FCS experiments, the autocorrelation amplitude G(*t*) is inversely proportional to the number of molecules in the detection volume. In case of a high number of molecules in the confocal volume, the fluctuation amplitudes are too low and detection noise compromises the calculation of the autocorrelation functions 29. On the other hand, an important factor for the applicability of assays and technologies in drug screening and intracellular analyses of molecular dynamics and distribution is the range of working concentrations of molecules such as FFPs. In drug discovery applications, initial screening of compounds binding to a cellular target requires a micromolar concentration range, whereas further identification of highly active binders needs nanomolar concentrations, which is well in the range of FCS applications 11, 25, 30, 31.

The expression levels of proteins in cell lines and primary cells or tissues of organisms vary and are differentially controlled due to cell‐specific expression of transcription factors and their posttranslational modifications as well as additional factors such as small noncoding RNA 32, 33, 34, 35, 36, 37, 38, 39, 40. For cell biological experiments, it has become of interest to achieve precise expression levels for recombinant proteins in cells 41, 42. The strength of the well‐established herpes simplex virus (HSV) thymidine kinase (TK) promoter has been shown to be in general weaker than that of other widely used viral promoters, the strong CMV and SV40 promoters 33, 34, 38, 42, 43. Studies have shown that the TK promoter contains a TATA box, a CCAAT box, and two SP1 elements (32, 33, 34, 35, 67, 68, 69 reviewed in 47). They are important to modulate the expression of viral genes and are bound by cellular transcription factors 32, 33, 34, 35, 36, 37, 38, 39, 40. In addition, the region of the 5′ untranslated region (5′‐UTR) of the HSV TK gene may also be involved in the regulation of transcription of the TK gene (33, 34, 46, 48 summarized in 47).

In this study, we choose the TK promoter 33, 34 and created mutants thereof to express eGFP and mCherry proteins in four mammalian cell lines to optimize protein expression levels for FCS analyses. The mammalian cell lines, the human osteosarcoma cell line U2OS, the human cervical carcinoma cell line HeLa CCL‐2, mouse embryonic fibroblast cells NIH 3T3, and primary BALB/c mouse stem cells (MSCs) were selected as they have been commonly used for cell biological experiments. In summary, it was shown that these established and newly constructed expression vectors having TK promoter deletion mutants allow a wide range of protein expression levels, from very high to very low, in both human and mouse cells. The expression of eGFP and mCherry under the control of two promoter constructs, TK2ST and TKTSC, yielded fluorescent protein levels optimally suitable for FCS and FCCS studies. Interestingly, TKΔSS, a TK promoter mutant having all regulatory elements of the full‐length TK promoter but lacking nucleotides +5 to +27 of the TK gene 5′‐UTR, yielded protein expression levels similar to the strong CMV promoter in both human cell lines and even higher protein levels in NIH 3T3 cells. Similarly, TK promoter constructs lacking these nucleotides of the TK gene 5′‐UTR yielded significantly higher protein expression levels than the equivalent constructs having the wild‐type 5′‐UTR sequence. These results suggest that a negative regulatory element is localized within the 5′‐UTR of TK gene regulating protein expression in cells, which are not infected by HSV.

## Results and Discussion

### HSV thymidine kinase promoter and its deletion mutant constructs for ectopic expression of fluorescent proteins in mammalian cells

The use of fluorescent proteins has been a game‐changer for modern cell biology and animal studies 1, 2, 3, 5, 6. GFP and its derivatives as well as the different flavors of fluorescent proteins in the red and infrared spectrum of light have been shown to interfere only minimally with cellular processes, although high expression levels of these fluorescent proteins fused to cellular proteins may contribute to artifacts in imaging techniques and other biological experimental approaches 5. A recent example that even slight overexpression of a protein or a fluorescent fusion protein may result in metabolic stress was demonstrated for Cdc45 49. In addition, quantitative microscopy techniques such as FCS and FCCS are sensitive to high levels of FP and FFP expression 14. As described above, FCS measurements require that FP and FFP concentrations are low enough that only few molecules are simultaneously detected in the observation volume 14, 25. Although there is not a strict limit for the maximum number of molecules per observation volume, experience suggests that five to twenty molecules per observation volume are optimal for FCS experiments. These levels are far below those levels normally achieved with commonly used CMV and SV40 promoters. Thus, the adjustments of the FP and FFP expression levels are necessary for cell and systems biology investigations relying on FP and FFP expression in eukaryotes.

**Figure 1:**
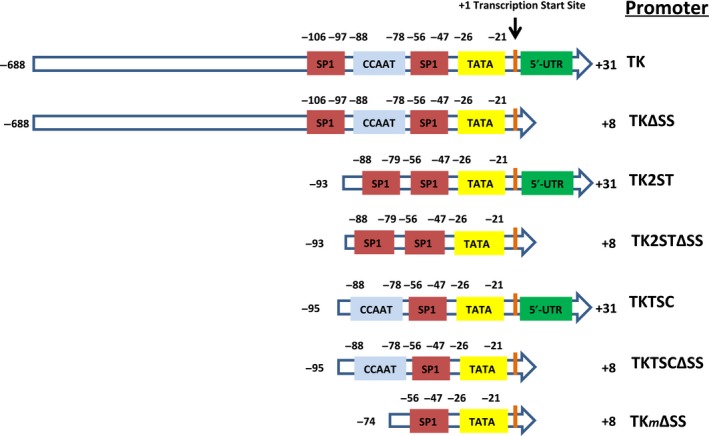
A schematic overview of the HSV TK promoter and its deletion mutants. The TK promoter and its derivatives were cloned into the plasmids pGW‐eGFP and pGW‐mCherry. TK, TKΔSS, TK2ST, TK2STΔSS, TKTSC, TKTSCΔSS, and TK *m*ΔSS represent the TK promoter constructs studied including the full‐length HSV TK promoter abbreviated as ‘TK’. Previously characterized transcription factor‐binding sites (SP1, CCAAT, and TATA box 33, 48) are highlighted within the schematic promoter presentation. The TK promoter sequences used upstream and downstream of the transcription start site are shown as numbers on the left and right of each sequence. To simplify the description, we introduce the following nomenclature as shown on the far right: TK, full‐length HSV thymidine kinase promoter; TK2ST, the promoter with two SP1 plus a TATA box but no CCAAT; TKTSC, the sequence with one SP1, and one TATA plus one CCAAT box; TK *m*ΔSS, the minimal TK promoter containing an SP1 and TATA box only with a deletion within the 5′‐UTR shortly after the viral TK gene transcription start site. ΔSS indicates sequences in which nucleotides +5 to +27 of the 5′‐UTR of the HSV TK gene were deleted in the specified promoter constructs (Fig. S1 and Tables for promoter sequence details).

To reduce levels of recombinant protein expression in comparison with the commonly used CMV and SV40 promoters, the TK promoter was used in the current study because the latter has been described as weaker promoter than CMV and SV40 promoters 43. In addition, we designed deletion mutants of the TK promoter with the aim to reduce levels of fluorescent protein expression in cells to achieve the necessary low FP and FFP levels for FCS studies. On the other hand, to simplify the exchange of coding sequences in these vectors and to allow recombination‐based cloning, we established these eGFP‐ and mCherry‐tagging expression vectors on the GW series of plasmids (Thermo Fisher Scientific, Dublin, Ireland). The GW system uses a site‐specific recombination reaction and permits easy assembly of a variety of expression vectors in a single step. In our case, we designed the destination vectors to contain the eGFP or the mCherry fluorescent protein with a GW cassette at the 3′‐end of the FP coding sequence. First, PCR was performed to delete 5′ and 3′ sequences of the TK promoter and parts of the 5′‐UTR of the TK gene (Fig. 1; Fig.S1 ; Tables S1and S2). In addition, flanking *Ase I* and *Nhe I* restriction sites were introduced to allow the introduction of the TK promoter using *Ase I* and *Nhe I* restriction sites in the GW vectors containing eGFP and mCherry to replace the CMV promoter sequences. Thus, new destination vectors were produced that express eGFP‐ and mCherry‐tagging proteins under the control of the full‐length TK and TK deletion mutants (Fig. 1).

The TK promoter is well studied, and it has been shown to rely primarily on CCAAT, SP1, and TATA boxes adjacent to the transcription start site to control protein expression, which form the TK basal promoter and are found within about 110 base pairs upstream of the transcription initiation site [32, 33, 34, 45, 46, 47, 48, 50. Therefore, we focused on this region including or excluding the TK gene 5′‐UTR to design deletion mutants creating a collection of TK gene promoter mutants, which includes ‘TKTSC’ (shortened TK promoter with only TATA, SP1, and CCAAT elements, promoter region ‐93 to +31) and ‘TK2ST’ (TK promoter deletion mutant with only two SP1 elements and a TATA box but without the ‘CCAAT’ element). As it was reported that in HSV‐infected cells, a linker scanning (LS) mutant, LS +5/+15, within the 5′‐UTR of the TK gene directly adjacent to the transcription start site reduces TK protein expression, the deletion mutant ‘TKΔSS’ (‐689 to +8) was created, which resembles the full‐length TK promoter (689 nucleotides upstream of transcription start site (nomenclature ‐689) to position 31 of the transcribed 5′‐UTR (nomenclature +31)) but lacks the TK gene 5′‐UTR sequence nucleotides +5 to +27. Similarly, ‘TK2STΔSS’ and ‘TKTSCΔSS’ are variants of TK2ST and TKTSC, respectively, which lack the nucleotides +5 to +27 of the 5′‐UTR of the TK gene (Fig. 1; Fig. S1 and Table S2). In addition, the TK promoter deletion mutant ‘TK*m*ΔSS’ was constructed, which only consists of SP1 and TATA elements and does not have most of the sequence of the 5′‐UTR of TK gene (nucleotides +5 to +27).

### Expression of fluorescent proteins using viral promoters

The destination vectors containing the fluorescent proteins, eGFP and mCherry, under the control of TK promoter and its deletion mutants were tested for protein expression in two human cell lines, HeLa CCL2 and U2OS, and two mouse cell lines, NIH 3T3 and primary BALB/c MSC. The FP levels were then compared to the FP expression levels controlled by the CMV and SV40 promoters.

**Figure 2:**
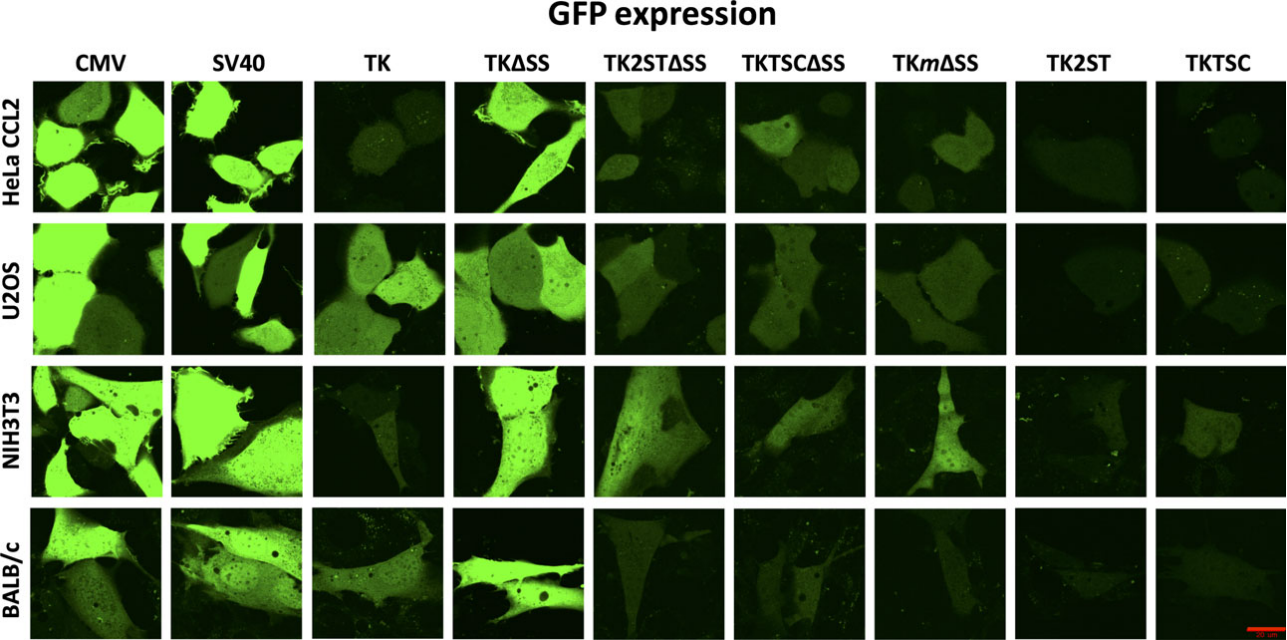
Confocal laser scanning microscopy images of eGFP expressed using a variety of promoter sequences. Rows (panels top to bottom) display various cell types studied: human HeLa CCL2 cells, human U2OS cells, mouse NIH 3T3 cells, and mouse stem cells, BALB/c, as indicated. Columns represent promoter sequences used in the experiments. They are marked as CMV, SV40, and TK, and the deletion mutants of the latter (TKΔSS, TK2STΔSS, TKTSCΔSS, TK *m*ΔSS, TK2ST, and TKTSC; ΔSS indicates sequences, in which nucleotides +5 to +27 of the 5′‐UTR of the HSV TK gene were deleted). Images shown are representative of multiple independent experiments (at least three independent transfections) and show the eGFP fluorescence levels of three different viral promoters, CMV, SV40, and TK. In addition, TK promoter deletion mutant constructs established in this study are also shown for comparison. Most mammalian cells show a very high expression of eGFP protein when transfected with plasmids containing CMV and SV40 promoters (compare the first two columns of all four mammalian cell lines). In contrast, the expression levels of full‐length TK promoter varied depending on the cell line (third column). The deletion mutants TK2STΔSS, TKTSCΔSS, and TKmΔSS (columns 4 to 7) always showed low expression of eGFP, but these expression levels are still on the high side for FCS experiments (data not shown). Constructs TK2ST and TKTSC showed optimal fluorescence for FCS studies (columns 8 and 9, respectively). The lower right image contains the scale bar = 20 μm valid for all other images. The images present cells with representative fluorescent protein expression levels for each promoter construct and cell line. To make the intensities of fluorescent proteins comparable, the images were taken with the same parameters including amplification settings of the detectors and the later handling of the images for all promoter constructs and cell lines. Therefore, the intensities of GFP for CMV, SV40, and TKΔSS promoter constructs are very high, whereas the fluorescent signals seemed to be barely detectable for TK2ST and TKTSC promoter constructs. It is important to note that the protein levels expressed under the control of these two promoters are optimal for FCS as discussed in the main text.

**Figure 3:**
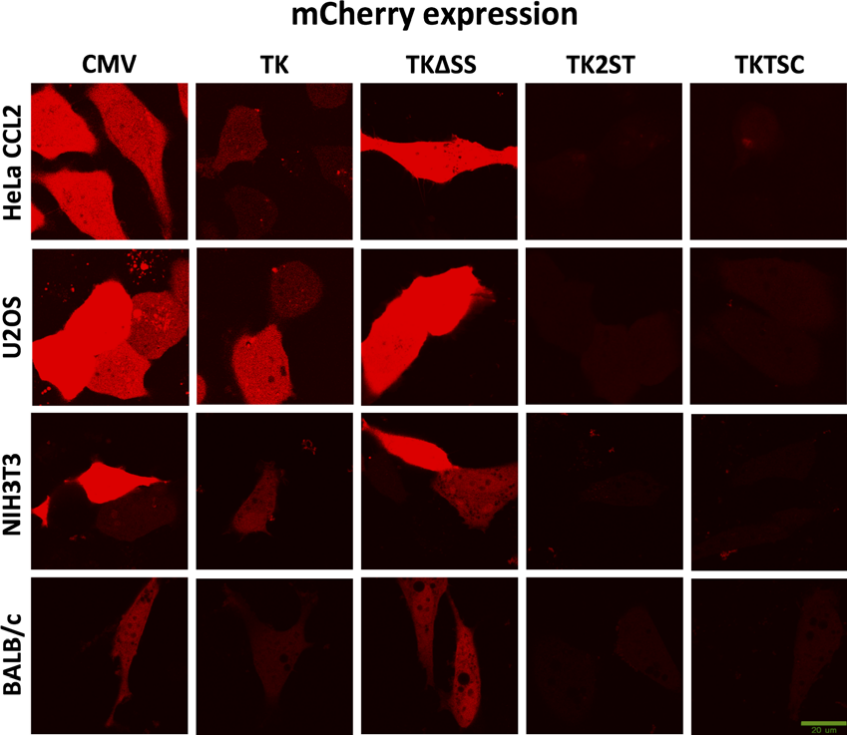
Confocal laser scanning microscopy images of mCherry expressed using a variety of promoter sequences. Rows, panels 1 to 4 (top to bottom), display the expression of mCherry in the four cell lines, human HeLa CCL2, human U2OS, mouse NIH 3T3 cells and mouse stem cells BALB/c, as indicated after the transfection with the indicated expression vectors. Columns present promoter sequences controlling expression of mCherry as shown, which are in detail described in Fig. 2. Fluorescent microscope images show comparison of mCherry fluorescence expressed by different promoters and deletion mutant constructs described in this study. Most of the cells show very high expression of mCherry protein when transfected with plasmids containing CMV and TKΔSS promoters. The full‐length TK promoter shows a reduced but still high expression level, whereas constructs TK2ST and TKTSC show optimal fluorescence for FCS studies. Images shown are representative of multiple experiments (at least three independent transfections). The lower right image contains the scale bar = 20 μm valid for all other images. The images present cells with representative mCherry expression levels for each promoter construct and cell line. To make the intensities of mCherry comparable, the images were taken with the same parameters including amplification settings of the detectors and the later handling of the images for all promoter constructs and cell lines. Therefore, the intensities of red fluorescence for promoter constructs CMV and TKΔSS are very high meaning high mCherry expression levels. In contrast, the fluorescent signals seemed to be barely detectable for TK2ST and TKTSC promoter constructs, but both these mCherry levels are optimal for FCS as discussed in the main text.

**Figure 4:**
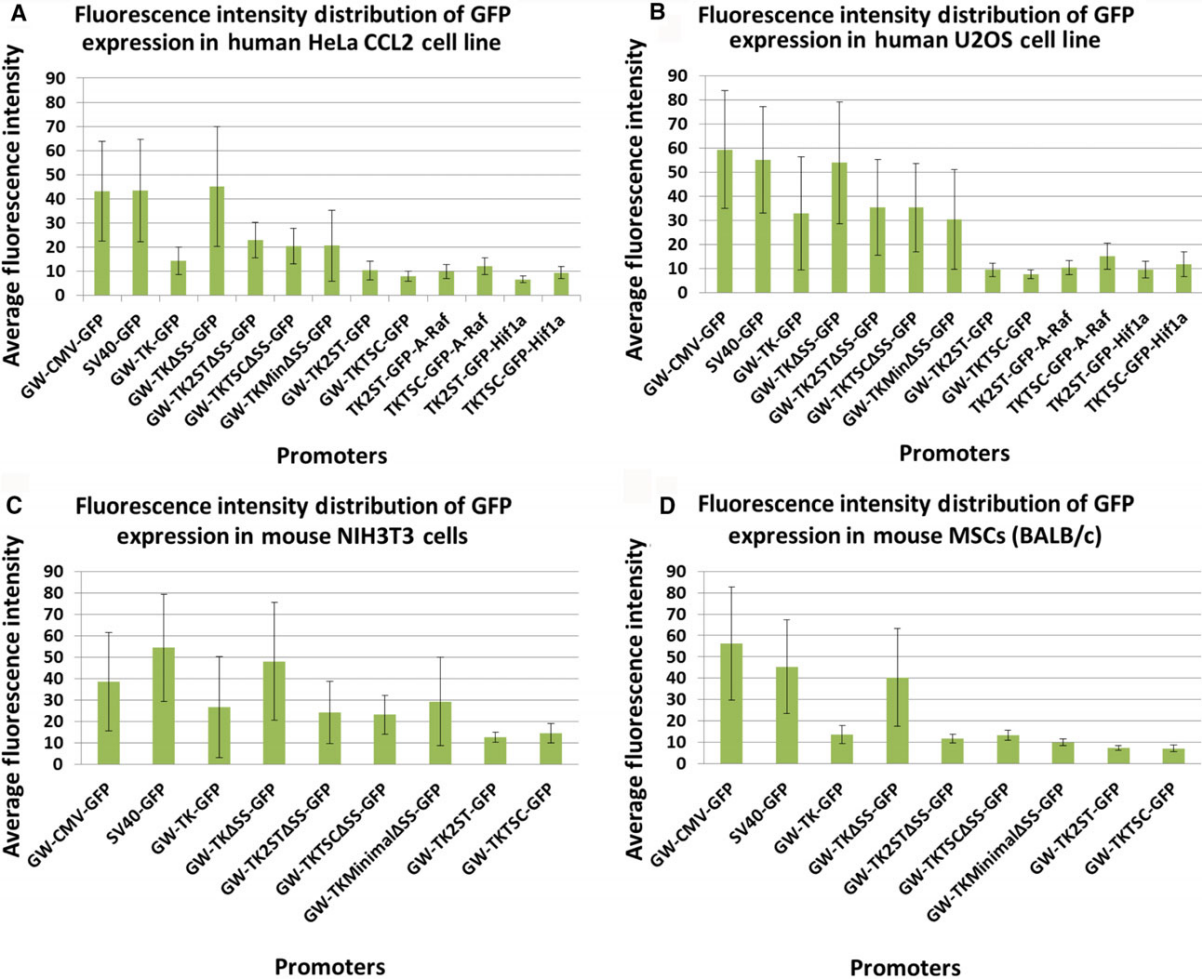
Quantitative comparison of eGFP expression under the control of the TK promoter and derivatives thereof in human and mouse cells. Fluorescence intensities were measured using the ImageJ software to compare eGFP expression levels under the control of the TK promoter and derivatives thereof with other viral promoters such as CMV and SV40 in human and mouse cells (panel A: HeLa CCL2 cells; panel B: U2OS cells; panel C: NIH 3T3 cells; and panel D: primary MSCs (BALB/c)). In addition, the expression of eGFP fusion proteins with A‐Raf and Hif1α (TK2ST‐GFP‐A‐Raf, TKTSC‐GFP‐A‐Raf, TK2ST‐GFP‐Hif1a, and TKTSC‐GFP‐Hif1a) under the control of the TK2ST and TKTSC promoter constructs was performed and quantified in parallel. Fluorescence intensity measurements show that the deletion mutants TK2ST and TKTSC have a significantly lower intensity in comparison with CMV, SV40, full‐length HSV TK promoter (TK), and the TK deletion construct TKΔSS. Transfection experiments were performed in duplicate, and at least *n* = 10 fluorescent cells were selected for each measurement. The results represent the average and the standard deviation of the fluorescence intensities. The intensity of the construct TK2ST‐GFP in HeLa CCL2 cells (panel A) was arbitrarily set to 10. The highest expression levels of the CMV, SV40, and TKΔSS promoter constructs were out of range for fluorescence intensity measurements, and therefore, only the low and intermediate expression levels could be quantified for these cells.

**Figure 5:**
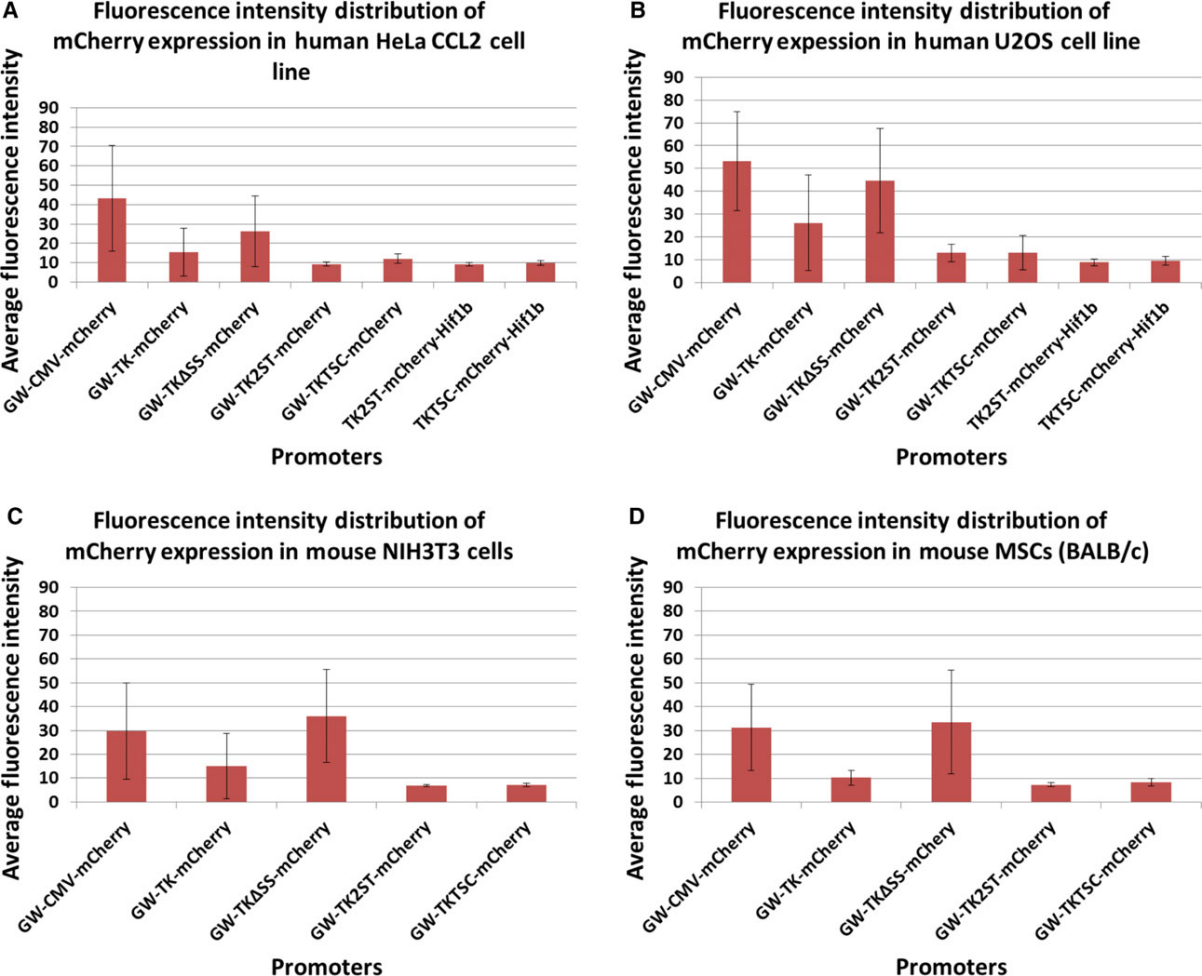
Quantitative analysis of mCherry expression in human and rodent cells. The fluorescent protein mCherry was expressed under the control of the CMV promoter, the full‐length TK promoter, and derivatives thereof as indicated and tested in human and mouse cells (panel A: HeLa CCL2 cells; panel B: U2OS cells; panel C: NIH 3T3 cells; and panel D: primary MSCs (BALB/c)). In addition, two promoter constructs to express the protein Hif1β as an mCherry fusion protein (TK2ST and TKTSC; TK2ST‐mCherry‐Hif1b and TKTSC‐mCherry‐Hif1b) were tested in these cells. Fluorescence intensities were measured using the ImageJ software. These measurements show that the TK promoter deletion mutants TK2ST and TKTSC have a significant decrease in intensity as compared to CMV, the full‐length TK promoter, and TKΔSS deletion construct. Experiments were performed in duplicate, and at least *n* = 10 fluorescent cells were selected for each measurement. The results represent the average and the standard deviation of the fluorescence intensities. The highest expression levels of the CMV and TKΔSS promoter constructs were out of range for fluorescence intensity measurements, and therefore, only the low and intermediate expression levels could be quantified for these cells.

After transient transfection, plasmids containing CMV and SV40 promoter constructs showed heterogeneity in fluorescent protein expression levels, which is evident in all cell populations tested (Fig. 2—the two left columns show a selection of reproducible fluorescence microscopy images of eGFP expression driven by the CMV and SV40 promoters, respectively; Fig. 3—the far left column shows a selection of reproducible fluorescence microscopy images of mCherry expressions, which served as examples for the CMV promoter‐dependent expression levels). In addition, a considerable amount of overexpression of these fluorescent proteins was determined, which makes the expression quantification difficult for the analysis by FCS microscopy techniques. Although DNA amounts were varied to overcome the FP overexpression by CMV and SV40 promoters, FPs’ overexpression levels in cells with low DNA concentrations were not reduced; instead, lower numbers of transfected cells were observed (data not shown). In contrast, the full‐length TK promoter and its deletion mutants TK2STΔSS, TKTSCΔSS, and TK*m*ΔSS showed low expression levels of eGFP and mCherry in comparison with the CMV and SV40 promoters (in Fig. 2; compare fluorescence microscopy pictures in columns 3, 5, 6, and 7 with columns 1 and 2, respectively; in Fig. 3, compare pictures in column 2 of the full‐length TK promoter with column 1, the CMV promoter; their quantifications are summarized in Figs 4 and 5). However, it is worth to note that these expression levels of eGFP and mCherry of the full‐length TK promoter and its deletion mutants TK2STΔSS, TKTSCΔSS, and TK*m*ΔSS were still at higher concentration levels and it was difficult to perform FCS analyses with these levels of ectopically expressed fluorescent proteins especially in U2OS cells (Figs 2 and 3, second panels from the top, and data not shown).

Therefore, additional TK promoter deletion mutants were tested to find the optimal promoter sequences to carry out FCS measurements in cells with low ectopic expression levels. To reduce expression levels, TK promoter constructs lacking the single CCAAT box or one of the two SP1 boxes (named TK2ST and TKTSC, respectively) were established by site‐directed mutagenesis and cloned upstream of the eGFP and mCherry coding sequences (Fig. 1 andSupplementary Material). In comparison with the other promoter constructs, deletion mutants TK2ST and TKTSC exhibited minimal fluorescence of the expressed fluorescent proteins (Fig. 2, columns 8 and 9, respectively; Fig. 3, columns 4 and 5, respectively). The low expression levels make these promoter constructs less favorable for image acquisition, but these expression levels of the fluorescent proteins are optimally suitable for FCS studies as described below.

Interestingly, the mutant TK promoter with a deletion within the 5′‐UTR, TKΔSS, showed in qualitative image analyses an expression level of eGFP and mCherry comparable to CMV and SV40 promoters (Fig. 2, compare column 4 labeled as TKΔSS, with columns 1 and 2 representing CMV and SV40 promoter‐controlled expression levels, respectively; Fig. 3, compare column 3, indicated as TKΔSS, with column 1, highlighted as CMV). This relatively high expression level of eGFP and mCherry was confirmed in quantitative analyses (Figs 4 and 5). In addition, an increase in protein expression levels was determined for all constructs with this sequence deletion within the TK gene 5′‐UTR. These deletion mutants, TK2STΔSS and TKTSCΔSS, also showed higher protein expression levels than the equivalent deletion mutants TK2ST and TKTSC in all four cell lines tested (Fig. 2 for a qualitative analysis—compare the columns shown as TK2STΔSS and TKTSCΔSS with those labeled as TK2ST and TKTSC; and Fig. 4 for a quantitative comparison). These data suggest that protein expression under the control of TK promoter does not require all sequences downstream of the TATA box and that an inhibitory activity might be associated with this region.

The importance of 5′‐UTR sequences within the HSV TK promoter was previously recognized using linker scanning mutants 45, but the molecular mechanism is still unclear 47. The mutant LS +5/+15 shows a reduction in mRNA and protein synthesis of HSV‐infected primate cells but in neither HSV‐infected mouse L cells nor Xenopus oocytes 45, 48, 51. It has been shown that the HSV protein ICP4 binds to the 5′‐UTR region via a noncanonical ICP4‐binding site and enhances TK gene expression by about an order of magnitude, but the mutation of the sequence +5 to +15 (LS +5/+15) does not interfere with the ICP4 binding, and most likely its interactions with TBP are important for the ICP4‐dependent regulation of TK expression in HSV‐infected primate cells (32, 33, 34 summarized in 47). Thus, proteins binding to the region +5 to +15 of the 5′‐UTR‐coding DNA might not be important for the regulation of HSV TK expression. Recently, it was shown that the cellular RNA helicase DDX3X is important for the regulation of HSV early genes including UL23, which encodes for HSV TK, suggesting that TK mRNA structures or small RNA may regulate HSV TK gene expression in HSV‐infected cells 52. It is important to note that none of the cell lines studied here expresses ICP4 and the putative factor binding to the region +5 to +27 of the 5′‐UTR at the DNA or RNA level seems to act as a repressor and not an activator in uninfected mouse and human cells as removing its putative binding site increases protein expression. On the other hand, the region could be involved in mRNA stability or translation control including its binding to a cellular miRNA. Thus, removing the region interferes with protein expression 53, 54. This hypothesis would also be consistent with the stimulation of HSV TK gene expression by the cellular RNA helicase DDX3X 52. These assumptions are not in contradiction to the lack of reactivity of the LS +5/+15 mutant in HSV‐infected mouse L cells as the study only looked at TK mRNA not the protein levels 51.

Previously, it has been shown that TK promoter does not exhibit an equal strength in all cell lines and that the presence of GATA transcription factors could interfere with the expression efficiency of genes controlled by the TK promoter 55. However, the GATA transcription factor‐binding site is found ‐249 to ‐255 upstream of the TK full‐length and TKΔSS but not in the deletion mutants TK2ST and TKTSC and its derivatives TK2STΔSS and TKTSCΔSS (Fig. S1 ; Tables S1 and S2). Therefore, this transcription factor cannot be responsible for the differences in protein levels ectopically expressed using these vectors. It also has been shown that other cellular transcription factors such as the VDR/RXR (vitamin D receptor/retinoid X receptor) may bind VDR/RXR binding site near the TK promoter TATA box and modulate the transcription activity of the TK promoter in a cell line‐dependent manner 56. The latter is still present in these ‘ΔSS’ deletion constructs and therefore would not be responsible for the increase in protein expression. However, this could still mean that additional yet unidentified cellular factors, which are present in human and mouse cells, are responsible for the decrease in expression of these fluorescent proteins.

The influence of the 5′‐UTR on protein expression in eukaryotes has recently gained significant interest and has been extensively studied in yeast and mammalian cells 57, 58, 59. Secondary structures, an out‐of‐frame upstream AUG (yielding a wrong shorter protein), and the last three nucleotides upstream of the open reading frame (ORF) have been found to be the strongest indicators for protein‐level variations and are suggested to have the most impact on the expression levels of a variety of proteins 58, 59. None of the 5′‐UTRs used here coded for an out‐of‐frame upstream AUG and the three nucleotides upstream of the start ATG were not changed in the experiments presented here (Fig. S1) leaving the possibility of secondary structures in the transcribed RNA being involved in regulating protein expression. The 5′‐UTR of the HSV TK gene merged to the eGFP and the mCherry ORF is GC‐rich (70%; Fig. S1). Using published algorithms accessible on the University of Rochester and Vienna RNA structure webservers, a relatively strong hairpin structure with a free energy of −16.3 kcal·mol^−1^ for the full‐length 5′‐UTR was predicted (Fig.S2) 60, 61, 62. In contrast, the deletion in the 5′‐UTR yielded a smaller putative hairpin with a predicted free energy of −5.8 kcal·mol^−1^ (Fig. S2B), suggesting that secondary structures within the 5′‐UTR may have an influence on the gene expression under the control of TK promoter. Alternatively, factors may bind to this putative hairpin of the TK 5′‐UTR and influence protein translation 63. These findings suggest that posttranscriptional control of genes expressed under the control of the HSV TK promoter including its 5′‐UTR may have an important role on top of the binding and regulation via transcription factors binding to the promoter sequences. Interestingly, the sequence introduced by the mutation LS +5/+15 is very GC‐rich 45, 48, 51 and increases the stability of a putative hairpin (data not shown). Thus, the introduction of the mutation LS +5/+15 may increase the strength of an existing repressor activity and not diminish the activity of a transcription factor‐binding site to stimulate transcription of the TK gene. However, further research is required to understand how deletions within the TK gene 5′‐UTR lead to increased expression levels of this commonly used promoter. These findings presented here could be of general interest as the expression of HSV TK in cancer cells has been recently exploited in strategies for cancer therapy 64. Moreover, the reactivation of silent HSV copies in nerve cells is a common occurring health risk and the expression of HSV TK is crucial for the reactivation of HSV in human nervous cells 65.

### TK promoter deletion mutants show low levels of protein expression in mammalian cells

These preliminary qualitative studies were followed by quantitative image analysis to allow a ranking of the promoter strength and to select the best promoter constructs for FCS. At least 10 cells were selected in each case to determine the average fluorescence intensity. It is important to mention here that especially for the CMV, SV40, and TKΔSS promoters, only low‐ to medium‐expressing cells shown in Figs 2 and 3 were selected to avoid overexpression artifacts in image analysis (Figs 4 and 5). In the human cell lines tested, the CMV, SV40, and TKΔSS promoters fused to eGFP cDNA yielded similar expression levels (Fig. 4A,B), whereas the full‐length TK promoter was weaker than these three promoters (compare the third column in Fig. 4A,B with the indicated columns). Similarly, in the tested human cell lines the expression levels of mCherry controlled by CMV and TKΔSS promoters were very high, and the expression levels under the control of these promoter sequences were higher than those protein amounts, which were achieved using the full‐length TK promoter construct (Fig. 5A,B). In two mouse cell lines, similar results were obtained with the exemption of the CMV promoter construct, which shows a relatively low expression of eGFP and mCherry in NIH 3T3 (Figs 4 and 5, panel C). Contrarily, the high expression levels achieved with the TK promoter deletion mutant TKΔSS are reproducible with both the eGFP and mCherry expression vectors in mouse and in human cells (Figs 4 and 5, panels C and D). However, it is worth to note that the reduced expression levels of eGFP and mCherry under the control of the full‐length TK promoter were still at the high end of the concentration range to optimally perform FCS analyses especially in U2OS cells (Figs 2 to 5, and data not shown). In U2OS cells, the full‐length TK promoter construct still produced so much protein that it was difficult to find cells at the right concentration level for FCS (see below and data not shown).

**Figure 6:**
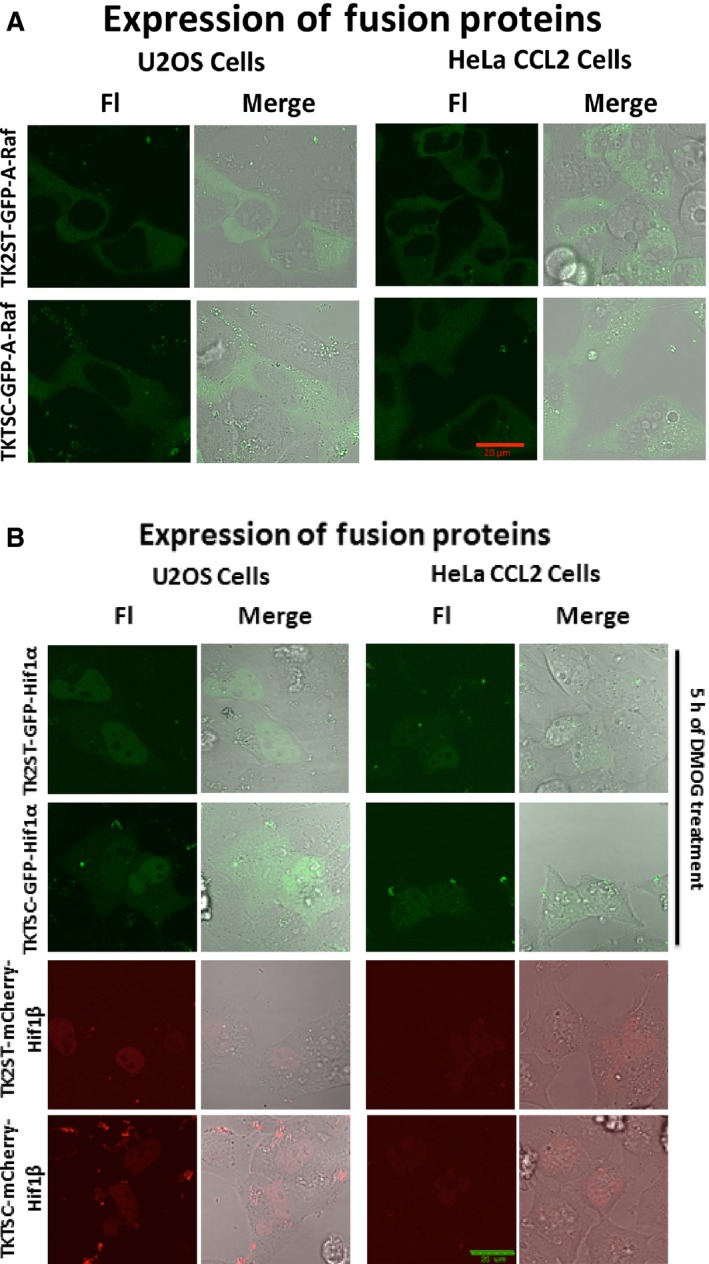
Confocal laser scanning microscopy images of eGFP and mCherry fusion protein expressions. (A) The first and third columns represent the confocal fluorescence microscopy detections (Fl), whereas the second and fourth columns show the merge of fluorescence microscopy and phase contrast images, respectively. U2OS and HeLa CCL2 cell lines show minimal expressions of A‐Raf protein presented in green, which were suitable for FCS experiments. The experiments were performed three times, and representative images are shown. U2OS and HeLa CCL2 cell lines showing minimal expression of eGFP‐A‐Raf fusion proteins using the promoter constructs TK2ST and TKTSC (panel A, first and second rows, respectively). Scale bar = 20 μm. (B) Confocal laser scanning microscopy images (Fl) of eGFP‐Hif1α (top two panels; cells were transfected with the expression plasmid for 24 h and then grown in the presence of 1 mm DMOG for 5 h) and mCherry‐Hif1β (two lower panels) expression experiments are presented using the TK2ST and TKTSC promoter constructs for the control of protein expression. The experiments were performed three times, and representative images are shown. The protein expression was under the control promoter constructs TK2ST (first and third rows) and TKTSC (second and third rows) U2OS and HeLa CCL2 cell lines showing minimal expression of eGFP‐Hif1α and mCherry‐Hif1β proteins, which were suitable for FCS experiments. Scale bar = 20 μm.

In contrast, the FP expression levels in cells transfected with plasmids carrying eGFP and mCherry expression under the control of newly established TK2ST and TKTSC promoters were consistently very low. The expression levels using these promoters were also more coherent in most cells using eGFP or mCherry as reporters (Fig. 4 and 5, respectively). It is important to note that a number of proteins tend to produce discrete foci within cells 66. We noticed that the fluorescence intensity was in general uniform throughout the cells except few scattered fluorescent foci which were present in Figs 2, 3, and 6 for both the TK2ST and TKTSC constructs. These foci are most probably microtubular organization centers or membrane ruffles as they are located mostly in the outskirts of the cells. These foci were part of the fluorescence intensity quantification data because the whole cell was selected as a region of interest during the quantification process. However, these foci were avoided during the FCS experiments because their high density of fluorescent proteins in a given area could interfere with the FCS measurements.

The transfection of HeLa CCL2, U2OS, and NIH 3T3 cells but not primary BALB/c cells with two eGFP expression constructs, TK2STΔSS and TKTSCΔSS, showed higher expression levels and higher degrees of variation than the TK2ST and TKTSC promoter sequences themselves (compare Fig. 4 panels A, B, and C with panel D). In summary, these results suggest that we were able to establish promoter constructs with low expression levels, which are optimal for FCS (see below). We cannot rule out that differences in transfection efficiencies contribute to the differences in expression levels observed for these constructs, although multiple DNA preparations and DNA amounts were used without changing the results (data not shown). Moreover, shortening the 5′‐UTR of these promoter sequences reproducibly yielded a significant increase in protein expression levels and fluorescence signals (Figs 2 and 4), which strongly suggests that transfection efficiencies were not responsible or not the main reason for the low expression levels seen. However, it is also important to mention that depending on the cell line, different promoter constructs might be optimally suitable for the FCS measurements, which could be tested in parallel using the ease of the Gateway® cloning system to establish expression constructs. Thus, the availability of promoters with various strengths based on the TK promoter (this paper) or on the CMV promoter 41 is a great advantage for researchers having to ectopically express genes to study the functions of their products in living cell without overexpressing them because the promoters as demonstrated here show higher and lower expression levels depending on cell lines.

### Cellular proteins fused to fluorescent proteins expressed at low levels using new promoter constructs

A major aim of modern cell biology is to analyze the function of proteins in cellular environments and to study them in living cells or even an organism as a whole. Here, the expression of fusion protein between cellular proteins and fluorescent proteins such as eGFP and its derivatives as well as those in the red and infrared spectrum has been very successful experimental tools. We tested a selected number of cDNA for their expression as fusion proteins with eGFP or mCherry in cells. Expression constructs of A‐Raf, Hif1α (hypoxia‐inducible factor 1), and Hif1β/AhR (aryl hydrocarbon receptor) were generated by transferring their ORFs from ORFEXPRESS™ Gateway^®^‐shuttle clones to destination vectors having TK promoter deletion mutants TK2ST and TKTSC driving their transcription in human cells (Table 1). In the following, U2OS and HeLa CCL2 cell lines were transfected with these expression vectors and showed minimal expression of A‐Raf, Hif1α, and Hif1β/AhR proteins in U2OS and HeLa CCL2 cell lines (compare Fig. 6A,B). Importantly, the expression constructs yielded fusion proteins localized in the equivalent compartments as the wild‐type A‐Raf, Hif1α, and Hif1β/AhR proteins 18, 67, 68, 69. Both eGFP‐A‐Raf expression constructs showed low levels of proteins in the cytosol (Fig. 6A) and the expression levels were in general comparable to the free eGFP (Fig. 4, panels A and B) although the expression of eGFP‐A‐Raf under the control of the TKTSC promoter was slightly increased.

**Table 1:** List of plasmids. In this study simian virus 40 (SV40) promoter, cytomegalovirus immediate‐early (CMV) promoter, and herpes simplex virus thymidine kinase (TK) promoter and deletion mutants thereof were used. TKΔSS, TK2ST, TK2STΔSS, TKTSC, TKTSCΔSS, and TK*m*ΔSS are deletion mutants of the TK promoter explained in detail in Fig. 1 and Fig. S1

Plasmid name	Promoter
pSV40‐eGFP	SV40
pGW‐CMV‐eGFP	CMV
pGW‐CMV‐mCherry	CMV
pGW‐TK‐eGFP	TK
pGW‐TK‐mCherry	TK
pGW‐TKΔSS‐eGFP	TKΔSS
pGW‐TKΔSS‐mCherry	TKΔSS
pGW‐TK2ST‐eGFP	TK2ST
pGW‐TK2ST‐mCherry	TK2ST
pGW‐TK2STΔSS‐eGFP	TK2STΔSS
pGW‐TKTSC‐eGFP	TKTSC
pGW‐TKTSC‐mCherry	TKTSC
pGW‐TKTSCΔSS‐eGFP	TKTSCΔSS
pGW‐TK*m*ΔSS‐eGFP	TK*m*ΔSS
pTK2ST‐eGFP‐A‐Raf	TK2ST
pTKTSC‐eGFP‐A‐Raf	TKTSC
pTK2ST‐eGFP‐Hif1α	TK2ST
pTKTSC‐eGFP‐Hif1α	TKTSC
pTK2ST‐mCherry‐Hif1β	TK2ST
pTKTSC‐mCherry‐Hif1β	TKTSC

The expression of eGFP‐Hif1α yielded an instable fusion protein (data not shown). The eGFP‐Hif1α fusion protein could only be detected after treating the transfected cells with 1 mm DMOG (dimethyloxalylglycine) to inactivate the cellular prolyl hydroxylases 70, 71 (Fig. 6B, top two panels). Thus, the addition of Hif1α to eGFP rendered the latter and produced an instable fusion protein, making the fusion protein an attractive model to study protein stabilization under hypoxic conditions. The fluorescence microscope analysis revealed that the stabilized eGFP‐Hif1α is mainly localized in the nucleus (Fig. 6B, top two panels). The expression levels of eGFP‐Hif1α in the presence of DMOG reached those of eGFP expressed without DMOG (compare Fig. 4A,B: GW‐TK2ST‐eGFP and GW‐TKTSC‐eGFP with TK2ST‐eGFP‐Hif1α and TKTSC‐eGFP‐Hif1α, respectively). Similarly, the mCherry‐Hif1β/AhR fusion protein was successfully expressed in human cells and the fusion protein mainly localized in the nucleus of the transfected cells (Fig. 6B, two lower panels). The fusion protein reached the same expression levels as the mCherry protein under the equivalent conditions (Fig. 5A,B). In summary, the measurement of the expression levels yielded that these fusion proteins under the control of the TK deletion mutant promoter constructs had levels comparable to those of the fluorescent protein expressed alone (Figs 4 and 5).

### Fluorescence correlation spectroscopy of fluorescent proteins expressed in human cells

Confocal microscope‐based FCS and FCCS are widely used techniques to quantitatively study mobility of molecules, number of molecules, and biomolecular interactions in solution, membrane, and living cells 19, 20, 21. For cellular studies, expressing fusion fluorescent proteins at lower level is difficult as the most widely used expression vectors contain CMV or SV40 promoter sequences, which mostly result in high protein expression levels that are not well suitable for FCS studies (Figs 2 and 3). The researchers often prebleach the cells to reduce the fluorescent concentration before performing FCS measurements, which may influence the cellular metabolism due to the high laser intensities used 72. Alternatively, only cells which have an accidentally low expression can be used for these spectroscopic analyses.

**Figure 7:**
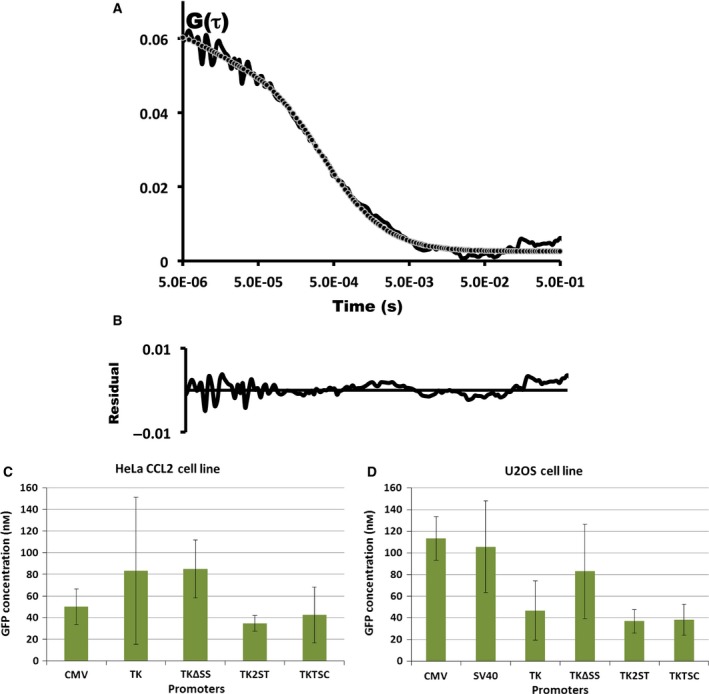
Fluorescence correlation spectroscopy with green fluorescent protein. (A) A typical autocorrelation curve of an FCS experiment using eGFP expression construct GW‐TKTSC‐eGFP as described above is presented. U2OS cells were transfected with GW‐TKTSC‐eGFP plasmid. After 24 h, confocal volume was positioned inside the cells, and eGFP fluorescence fluctuations were measured for 20 s. The recorded fluctuations were normalized using Eqn (1) in Materials and Methods, and the SymPhoTime software was used. The resultant autocorrelation function was fitted to one‐component three‐dimensional diffusion model and calculate diffusion time and number of molecules in the confocal volume. The black line corresponds to experimental FCS curve and dashed line represent corresponding diffusion model fit. In panel B, the presented curve corresponds to residual fit of experimental FCS curve and fitted model in panel A. Panels C and D show the concentration of eGFP (nM) derived from experimental determined number of molecules in the confocal volume for different pGW‐eGFP vector constructs, harboring the indicated promoters CMV, SV40, TK, TKΔSS, TK2ST, TK2STΔSS, and TKTSC, for transfection in HeLa CCL2 and U2OS cell lines, respectively. The eGFP concentrations were determined from at least 25 cells from different days for each construct and cell line. Error bars correspond to standard deviations of the experiments.

To overcome these problems, new GW‐expression vectors with full‐length TK and TK mutant promoter sequences were designed and established. The suitability of these newly constructed expression vectors were studied using FCS systems and we measured the diffusion time and calculated the eGFP concentration in different cell lines. Fig. 7A shows a typical autocorrelation curve of soluble eGFP expressed in U2OS cells. The observation volume defined by the confocal optics was positioned inside the cells. Fluorescence fluctuations arising due to eGFP molecules moving in and out of this volume were measured for 20 s and at different points within selected cells. The observation volume was calculated by measuring the diffusion time of 20 nm of Alexa Fluor 488 dye solution and found to be 0.5 fl. The autocorrelation curves were generated by normalizing the fluorescence fluctuations and fitted to one‐component three‐dimensional diffusion model, as described in 73. In Figure 7A, amplitude of the autocorrelation function G(*t*) corresponds to number of molecules in the observation volume because G(*t*) is inversely proportional to *N* (Eqn (2), Materials and Methods). The concentration of molecules in observation volume is calculated using the SymPhoTime software (PicoQuant GmbH, Germany) using the equation *C* = *N*/*V*_eff_ * *N*_A_, where *V*_eff_ is the measured confocal volume and *N*_A_ is the Avogadro number. The residual fit of experimental FCS curve and fitted model was in good agreement. The corresponding curve is presented in Fig. 7B, and the residual oscillates close to zero. The calculated diffusion coefficient of eGFP was 35.4 ± 4 μm^2^/s for all GW‐expression vectors and in different cell lines, which is similar to previously published values suggesting molecule is inert as expected 10, 17, 74.

The eGFP concentrations were determined in the transfected HeLa CCL2 and U2OS cells (Fig. 7C,D, respectively). Here, it is important to reiterate that FCS measurements in cells, which were transfected with vectors expressing protein under the control of the CMV, SV40, and TKΔSS promoters, could only be carried out after selecting cells with lowest expression levels. These cells were rare and the numbers of those cells were in the range of one percent of fluorescence‐positive cells in a given transfection experiment. In additional transfection experiments, in which the fluorescent proteins were expressed under the control of the full‐length TK promoter, both eGFP‐ and mCherry‐expressing cells showed a higher percentage of FCS‐suitable cells, but these cells still represented a relatively small subpopulation, whose size was dependent on the cell line analyzed (data not shown). As shown in Fig. 7 panels C and D, TK promoter‐transfected cells have wide distribution in the concentration of fluorescent molecules in both human cell lines. These findings indicate that a higher possibility and wider range of cells exist with low FCS‐suitable expression compared to CMV and SV40 promoters. However, in general, high expression levels of fluorescent proteins make it necessary to spend a significant amount of time to find cells that are suitable for FCS, and this process is frequently challenging.

In contrast, the eGFP expression with TK2ST and TKTSC showed the required low expression of eGFP in all cell lines tested and special selection of cells with low eGFP levels was not needed. The quantification of the eGFP molecules that were expressed using a selection of commonly available and newly constructed promoters showed that the eGFP concentrations in the analyzed cells were in the nanomolar (nm) range in both cell lines, HeLa CCL2 and U2OS (Fig. 7C,D, respectively). Both TK2ST and TKTSC deletion mutants show consistently low concentrations of eGFP molecules in the cell lines analyzed, and most importantly, neither cumbersome preselection nor prebleaching of cells was necessary.

As demonstrated, we have designed TK promoter deletion mutants with low protein expression level ideal for FCS and FCCS experiments. Together with the already existing and additional TK promoter sequences, a collection of promoters with a range of expression strength will allow expression of low to very low levels of proteins suitable for cell biology and systems biology analyses. The development of these TK deletion mutants will likely be able to eliminate overexpression‐based artifacts, which is important as previously demonstrated for eGFP‐Cdc45 fusion proteins 49, and support very sensitive fluorescence‐based and single‐molecule studies.

## Materials and methods

### Plasmids

A series of Gateway® (GW) Destination Vectors (Thermo Fisher Scientific) expressing eGFP and mCherry under the regulatory control of various promoter sequences were used (Table 1). The TK promoter was derived from pRL‐TK plasmid (Promega, Madison, WI, USA). An *Ase I* enzymatic restriction site was introduced at the 5′‐end and *Nhe I* site was introduced at the 3′‐end of the TK promoter fragment by PCR amplification using primers listed in Table 2 and sequences as presented in Fig. S1. PCR‐amplified sequences were introduced into pGEM‐T Easy vector system (Thermo Fisher Scientific), and the resulting promoter sequences were confirmed by restriction site analysis and sequencing (Eurofins Genomics, Germany). After sequence verification, TK promoter DNA fragments were introduced into the GW system to construct Gateway® Destination Vectors expressing eGFP and mCherry. The same procedure was applied to construct the TK deletion mutants.

**Table 2:** Primers used in this study to generate deletion mutants of TK promoter

Promoter	Primers 5′‐>3′
TK	ATTAATAAATGAGTCTTCGGACCTCGCG
	GCTAGCTTAAGCGGGTCGCTGCAGG
TKΔSS	ATTAATAAATGAGTCTTCGGACCTCGCG
	GCTAGCTCGGTGTTCGAGCCCACAC
TK2ST	GATTAATACAAACCCCGCCCGAATTCGAACACGCAGATGCAGTC
	GCTAGCTTAAGCGGGTCGCTGCAGG
TKTSC	GATTAATCGTCTTGTCATTGGCGAATTCGAACAC
	GCTAGCTTAAGCGGGTCGCTGCAGG

To generate expression clones, ORFs of A‐Raf (Cat. No. Z0699; GeneCopoeia™), Hif1α (Cat. No. GC‐T0096; GeneCopoeia™), and Hif1β/AhR (aryl hydrocarbon receptor; Cat. No. GC‐C0312; GeneCopoeia™) in ORFEXPRESS™ Gateway®‐shuttle clones were transferred to selected destination vectors (Table 1) using LR reaction (Gateway® Technology). All plasmid DNA were prepared using GenElute™ HP Plasmid Miniprep/Midiprep Kits (Sigma‐Aldrich, Germany). DNA concentrations of each plasmid DNA were independently assayed using spectrophotometry and analytical gel electrophoresis.

### Cloning

*E. coli* strains TOP10 and One Shot® *ccd*B Survival™ 2 T1R Competent Cells (Thermo Fisher Scientific) were used for cloning TK deletion mutants into GW vectors (17, 75 and van Vuuren, pers. communication). LB medium containing 50 mg·L^−1^ Kanamycin (Melford) was used for the growth of bacterial strains.

### Cell culture

The human osteosarcoma cell line U2OS, the human cervical carcinoma cell line HeLa CCL‐2, and mouse embryonic fibroblast cells NIH 3T3 were grown in Dulbecco's modified Eagle's medium (DMEM; Lonza) containing 10% fetal bovine serum (Sigma) and 100 units·mL^−1^ penicillin and streptomycin (both from Lonza) at 37 °C in a humidified incubator containing 5% CO_2_. Primary mouse BALB/c MSCs were cultured following the method of Peister *et al*. 76 and grown in α‐MEM (Gibco) containing 10% fetal bovine serum (Sigma) and 10% equine serum (Sigma). The animal work was carried out according to the institutional policy of best animal care practice. For live‐cell imaging and FCS, cells were seeded at the density of 10 000 cells/well in 8‐well chambered cover glass slide (Lab‐Tek; Thermo Scientific) and cultured for at least 18 h prior to transfection.

### Transfection

Cells were transfected with 100 ng of plasmid DNA per well using NanoJuice® transfection kit (Novagen) according to the manufacturer's instructions and left in the incubator for 24 h. Before the measurement, cells were washed two times using phenol‐red‐free DMEM (Lonza), and cells were left in phenol‐red‐free DMEM until the fluorescence measurements were performed.

### Live‐cell imaging and fluorescence correlation spectroscopy

The live‐cell confocal imaging and FCS measurements were carried out using FV1000 inverted epifluorescence microscope (Olympus, Hamburg) equipped with FCS module (PicoQuant GmbH, Berlin) and an UplanSApo 60 × water immersion objective lens (NA 1.2). The microscope chamber (PeCon GmbH) was kept at constant temperature of 37 °C and 5% CO_2_ humidified atmosphere. All experiments were performed with an excitation wavelength of 473 nm (Olympus, Hamburg) for eGFP and 550 nm for mCherry, and emitted fluorescence was collected back by the objective, was passed through appropriate dichroic mirrors and filters, with pinhole size of 100 μm, and directed to photomultiplier tube (PMT) for confocal imaging. Sampling speed of 20.0 μs/pixel and PMT voltage of 650V were used for all experiments. The laser intensity was kept at 1% (~ 2 μW) to prevent photobleaching and optimized for high‐quality imaging. The digital image output was 512 × 512 pixels with a 12‐bit resolution. In case of FCS, the light was directed to the single‐photon avalanche diode (MPD SPAD; Micro Photon Devices, Bolzano, Italy).

Data acquisitions and recordings were analyzed by a digital temporal correlator provided by the SymPhoTime software (PicoQuant GmbH, Berlin) to calculate the autocorrelation function G(τ), which represents the time‐dependent decay in fluorescence fluctuation intensity as in Eqn (1) 9.

(1) $$G\left(\tau \right)=\frac{\left\langle \delta F(\tau )\cdot \delta F(\tau +t)\right\rangle }{\left\langle F\left(t^{2}\right)\right\rangle }$$

In Eqn (1), G(τ) represents the <time average> of the change in fluorescence fluctuation intensity (δ*F*) at time (*t*) and later time (*t*+τ), divided by the square of the average fluorescence intensity (*I*).

Cells were imaged using confocal microscope and two random positions were selected inside the cells, and autocorrelation function was acquired for 20 s/run and each point was measured three times. The laser power was kept at 1.2 μW, as measured above the objective plane, and no significant changes in count rate due to photobleaching were observed. For each data set, we measured at least 25 cells on different days. Since the molecules confined inside certain compartments, those colliding with other molecules or organelles, or those being unable to diffuse freely inside the cells will lead to unusual variations or oscillations in the fluorescence fluctuation and these data were removed from the analysis. Autocorrelation curves measured from individual points were fitted by nonlinear fitting using the QuickFit software (http://www.dkfz.de/Macromol/quickfit/), and data were analyzed using one‐component three‐dimensional diffusion model considering triplet state of eGFP 17, 73.

(2) $$G\left(t\right)=\left[1+\frac{T}{\left(1-T\right)}\;\text{exp}\;\left(-t/\tau_{T}\right)\right]\cdot 1/N{\left(1+\frac{t}{\tau_{D}}\right)}^{-1}\cdot {\left(1+\frac{t}{S^{2}\tau_{D}}\right)}^{-1/2}$$

In Eqn (2), *T* denotes the fraction of fluorophores in the triplet state within the detection volume and τ_*T*_ is the lifetime of the triplet state, *N* is the average number of fluorescent molecules in the confocal volume, *t* is the lag time, and τ_*D*_ is the diffusion time 14. The analysis yields average dwell time of the fluorescent species within the observation volume (τ_*D*_), which is calculated from the midpoint of autocorrelation curve. The confocal volume is characterized by its lateral radius (ω) and its aspect ratio *S*. The lateral radius was calculated using 20 nm Atto488 organic fluorescent molecules (Atto GmbH, Siegen) in Milli‐Q H_2_O as described 5 and found to be 0.25 μm from known diffusion coefficient 10, 11, 31, 77. The diffusion coefficient (*D*) of fluorescent proteins and their fusion protein can then be calculated from the expression *D* = ω^2^/4τ_*D*_.

### Imaging data analysis

To determine the background contribution during live‐cell imaging, the fluorescence intensity of nontransfected cells (no fluorescent label) was measured and set as the background of these experiments. These background values were subtracted from all fluorescent intensity results carried out in parallel using ImageJ plugin. Background Subtract, Image Calculator, and Intensity Profile options in ImageJ (NIH, USA) were used for analyses to generate fluorescence intensity graphs in Figures 4 and 5.

## Author contributions

RA, SR, and HPN conceived and designed the project; RA and SR acquired the data; RA, SR, and HPN analyzed and interpreted the data; and RA, SR, FB, and HPN wrote the manuscript.

## Supporting information


**Fig. S1.** The HSV thymidine kinase promoter and its deletion mutants.
**Fig. S2.** RNA structure prediction of the 5′‐UTR of HSV thymidine kinase gene and its deletion mutant.
**Table S1.** Promoter Sequences.
**Table S2.** Promoter Sequences.
