# Supplementary material for: Optimizing fluorescent protein expression for quantitative fluorescence microscopy and spectroscopy using herpes simplex thymidine kinase promoter sequences
Source: FEBS Open Bio. 2018 May 8;8(6):1043–60. doi: 10.1002/2211-5463.12432 (PMC5985997; doi:10.1002/2211-5463.12432)
Supplement: Supplementary file 1 — Fig. S1. The HSV thymidine kinase promoter and its deletion mutants. Fig. S2. RNA structure prediction of the 5′‐UTR of HSV thymidine kinase gene and its deletion mutant. Table S1. Promoter Sequences. Table S2. Promoter Sequences. [file FEB4-8-1043-s001.pdf]

Supplementary Data.

Supplementary Figure 1: The HSV thymidine kinase promoter and its deletion mutants. The HSV thymidine kinase (TK) promoter was derived from the pRL-TK plasmid and deletion mutants were created. The promoter plus the respective 5’-UTR sequences and the multiple transcription factor binding elements are summarized. In the diagram the transcription start site at +1 and the sequences of the 5’ untranslated region (5’-UTR) are counted as +1 to +27 whereas the nucleotides upstream of the start site have a negative prefix. Bold and underlined nucleotides show the locations of the CCAAT box, TATA box, and the two Sp1 binding sites. The transcription start site is underlined and highlighted in red. The underlined nucleotides in the starting and ending of the whole sequence shows the introduced Ase I (TAAT) and Nhe I (CTAG) sites used for cloning. TK, TKΔSS, TK2ST, TK2STΔSS, TKTSC, TKTSCΔSS and TK*m*ΔS represent the established TK promoter constructs including the full length TK promoter, TK2ST, the promoter with two SP1 plus a TATTA box but no CCAAT, TKTSC, the sequence with one SP1, one TATTA plus one CCAAT box, and TK*m*ΔSS, the minimal TK promoter without viral transcription start site. Promoter sequences indicated as ΔSS have deleted the sequences +5 to +27 of the TK gene 5’-UTR.

Supplementary Figure 2: RNA structure prediction of the 5’-UTR of HSV thymidine kinase gene and its deletion mutant. The impact of the deletion in 5’-UTR of the HSV TK gene inspired a structural analysis of the RNA sequence using the established prediction algorisms available at the webservers of the University of Rochester and Vienna (Predict a Secondary Structure Web Server and RNAfold Web Server, respectively;

http://rna.urmc.rochester.edu/RNAstructureWeb/Servers/Predict1/Predict1.html;

http://rna.tbi.univie.ac.at/cgi-bin/RNAWebSuite/RNAfold.cgi) (1, 2). Both webservers predicted a relatively strong hairpin structure with a free energy of -16.3 kcal/mol for the full length 5’-UTR (panel 2A) whereas for the partial deletion of the 5’-UTR a smaller putative hairpin was predicted with free energy of -5.8 kcal/mol (panel 2B). The colour code indicates from purple to red a probability for the structure ranging from 0 to 1, respectively.

1. Gruber, A. R., Lorenz, R., Bernhart, S. H., Neubock, R. & Hofacker, I. L. (2008) The Vienna RNA websuite, *Nucleic acids research.* **36**, W70-4.
2. Reuter, J. S. & Mathews, D. H. (2010) RNAstructure: software for RNA secondary structure prediction and analysis, *BMC bioinformatics.* **11**, 129.

Supplementary Figure 1

Promoter	Sequence
**TK**	AseI Site<br>TAATCGGTGGTTAGGGTTTGTCTGACGCGGGGGGAGGGGGAAGGAACGAAACACTCTCATTCGGAGGCGGCTCGGGGTTTGGTCTTGGTG<br>GCCACGGGCACGCAGAAGAGCGCCGCGATCCTCTTAAGCACCCCCCCGCCCTCCGTGGAGGCGGGGGTTTGGTCGGCGGGTGGTAACTGG<br>CGGGTCGCTGACTCGGGCGGGTCGCGCGCCCCAGAGTGTGACCTTTTCGGTCTGCTCGCAGACCCCCGGGCGGCGCCGCCGCGGCGGCGA<br>CGGGCTCGCTGGGTCCTAGGCTCCATGGGGACCGTATACGTGGACAGGCTCTGGAGCATCCGCACGACTGCGGTGATATTACCGGAGACCT<br>TCTGCGGGACGAGCCGGGTCACGCGGCTGACGCGGAGCGTCCGTTGGGCGACAAACACCAGGACGGGGCACAGGTACACTATCTTGTCAC<br>CCGGAGGCGCGAGGGACTGCAGGAGCTTCAGGGAGTGGCGCAGCTGCTTCATCCCCGTGGCCCGTTGCTCGCGTTTGCTGGCGGTGTCCCC<br>GGAAGAAATATATTTGCATGTCTTTAGTTCTATGATGACACAAACCCCGCCCAGCGTCTTGTCATTGGCGAATTCGAACACGCAGATGCAGTC<br>1st Sp1 Binding Site CCAAT<br>+1 +31<br>GGGGCGGCGCGGTCCCAGGTCCACTTCGCATATTAAGGTGACGCGTGTGGCCTCGAACACCGAGCGACCCTGCAGCGACCCGCTTAGCTAG<br>2nd Sp1 Binding Site TATA Box Transcription Start Site → NheI Site
**TKΔSS**	AseI Site<br>TAATCGGTGGTTAGGGTTTGTCTGACGCGGGGGGAGGGGGAAGGAACGAAACACTCTCATTCGGAGGCGGCTCGGGGTTTGGTCTTGGTG<br>GCCACGGGCACGCAGAAGAGCGCCGCGATCCTCTTAAGCACCCCCCCGCCCTCCGTGGAGGCGGGGGTTTGGTCGGCGGGTGGTAACTGG<br>CGGGTCGCTGACTCGGGCGGGTCGCGCGCCCCAGAGTGTGACCTTTTCGGTCTGCTCGCAGACCCCCGGGCGGCGCCGCCGCGGCGGCGA<br>CGGGCTCGCTGGGTCCTAGGCTCCATGGGGACCGTATACGTGGACAGGCTCTGGAGCATCCGCACGACTGCGGTGATATTACCGGAGACCT<br>TCTGCGGGACGAGCCGGGTCACGCGGCTGACGCGGAGCGTCCGTTGGGCGACAAACACCAGGACGGGGCACAGGTACACTATCTTGTCAC<br>CCGGAGGCGCGAGGGACTGCAGGAGCTTCAGGGAGTGGCGCAGCTGCTTCATCCCCGTGGCCCGTTGCTCGCGTTTGCTGGCGGTGTCCCC<br>GGAAGAAATATATTTGCATGTCTTTAGTTCTATGATGACACAAACCCCGCCCAGCGTCTTGTCATTGGCGAATTCGAACACGCAGATGCAGTC<br>1st Sp1 Binding Site CCAAT<br>+1 +8 NheI Site<br>GGGGCGGCGCGGTCCCAGGTCCACTTCGCATATTAAGGTGACGCGTGTGGCCTCGAACACCGAGCTAG<br>2nd Sp1 Binding Site TATA Box Truncated Transcription Start Site →
**TK2ST**	-93 -81 ←<br>TAATACAAACCCCGCCCGAATTCGAACACGCAGATGCAGTCGGGGCGGCGCGGTCCCAGGTCCACTTCGCATATTAAGGTGACGCGTGTGG<br>AseI Site 1st Sp1 Binding Site 2nd Sp1 Binding Site TATA Box<br>+1 +31<br>CCTCGAACACCGAGCGACCCTGCAGCGACCCGCTTAGCTAG<br>Transcription Start Site → NheI Site
**TK2STΔSS**	-93 -81 ←<br>TAATACAAACCCCGCCCGAATTCGAACACGCAGATGCAGTCGGGGCGGCGCGGTCCCAGGTCCACTTCGCATATTAAGGTGACGCGTGTGG<br>AseI Site 1st Sp1 Binding Site 2nd Sp1 Binding Site TATA Box<br>+1 +8 NheI Site<br>CCTCGAACACCGAGCTAG<br>Truncated Transcription Start Site →
**TKTSC**	-95 -81 ←<br>TAATCGTCTTGTCATTGGCGAATTCGAACACGCAGATGCAGTCGGGGCGGCGCGGTCCCAGGTCCACTTCGCATATTAAGGTGACGCGTGT<br>AseI Site CCAAT 2nd Sp1 Binding Site TATA Box<br>+1 +31<br>GGCCTCGAACACCGAGCGACCCTGCAGCGACCCGCTTAGCTAG<br>Transcription Start Site → NheI Site
**TKTSCΔSS**	-95 -81 ←<br>TAATCGTCTTGTCATTGGCGAATTCGAACACGCAGATGCAGTCGGGGCGGCGCGGTCCCAGGTCCACTTCGCATATTAAGGTGACGCGTGT<br>AseI Site CCAAT 2nd Sp1 Binding Site TATA Box<br>+1 +8 NheI Site<br>GGCCTCGAACACCGAGCTAG<br>Truncated Transcription Start Site →
**TK*m*ΔSS**	-74 ← +1 +8 NheISite<br>TAATGAACACGCAGATGCAGTCGGGGCGGCGCGGTCCCAGGTCCACTTCGCATATTAAGGTGACGCGTGTGGCCTCGAACACCGAGCTAG<br>AseI Site 2nd Sp1 Binding Site TATA Box Truncated Transcription Start Site →

Supplementary Figure 2

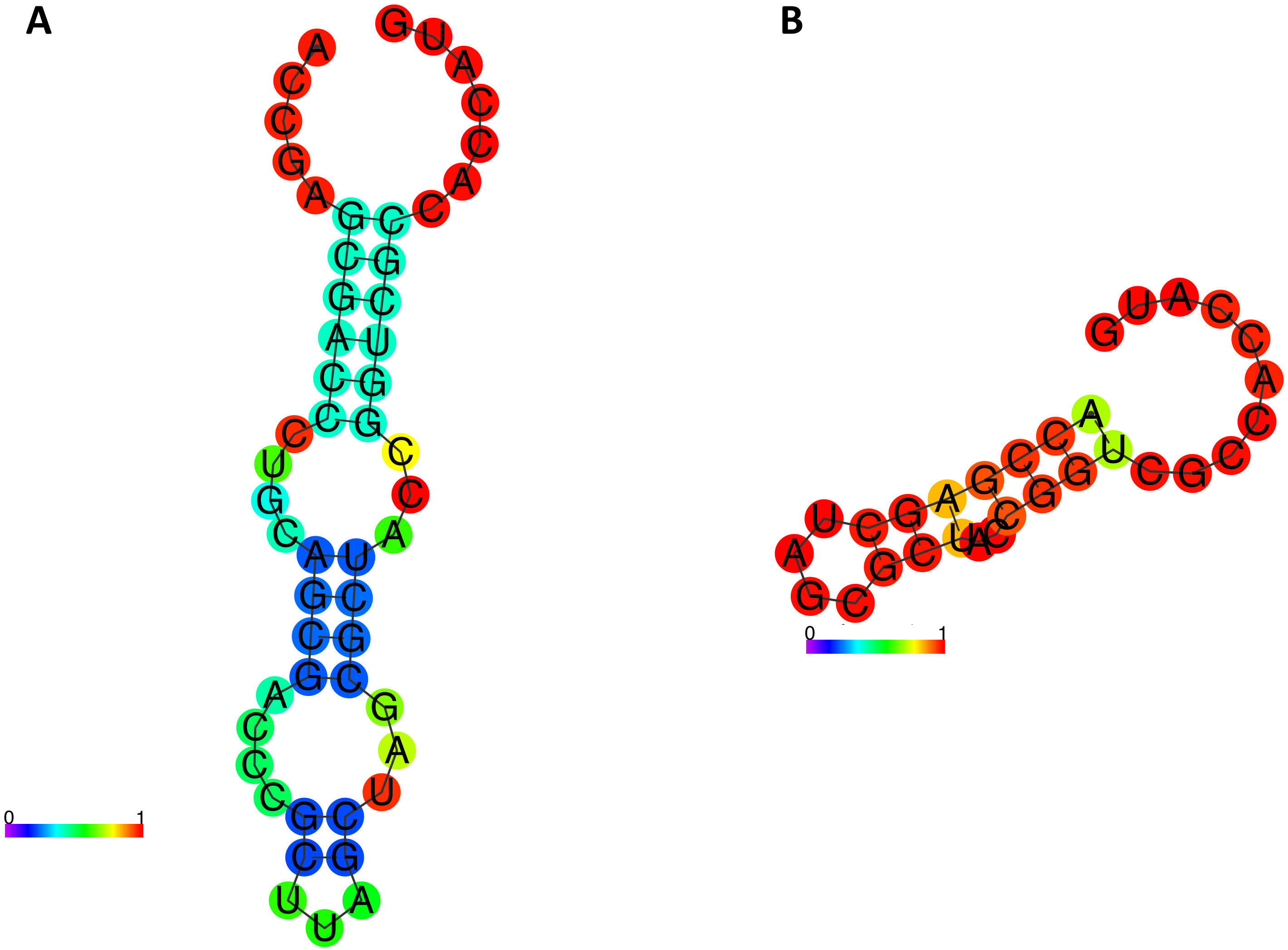

Supplementary Info.

- **Plasmid Maps**

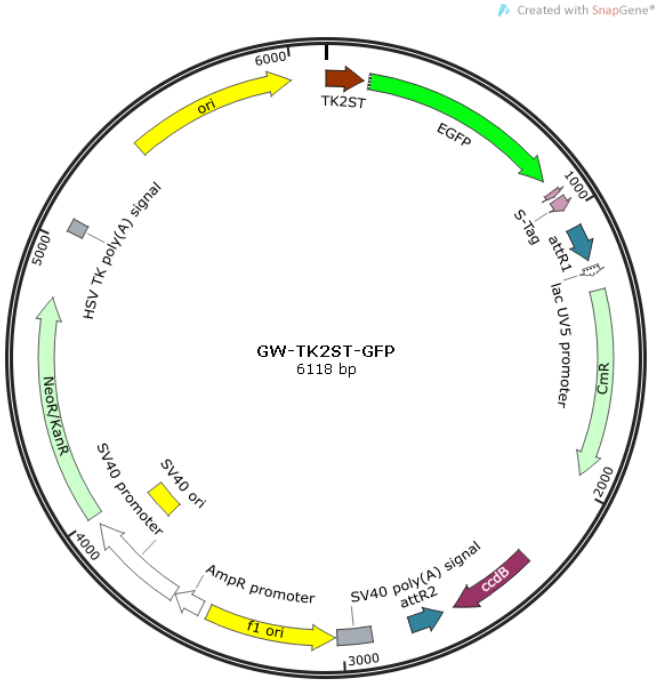
Created with SnapGene®
GW-TK2ST-GFP
6118 bp
TK2ST
EGFP
S-Tag
attR1
lac UV5 promoter
CmR
ccdB
attR2
SV40 poly(A) signal
f1 ori
AmpR promoter
SV40 promoter
SV40 ori
NeoR/KanR
HSV TK poly(A) signal
ori
1000
2000
3000
4000
5000
6000

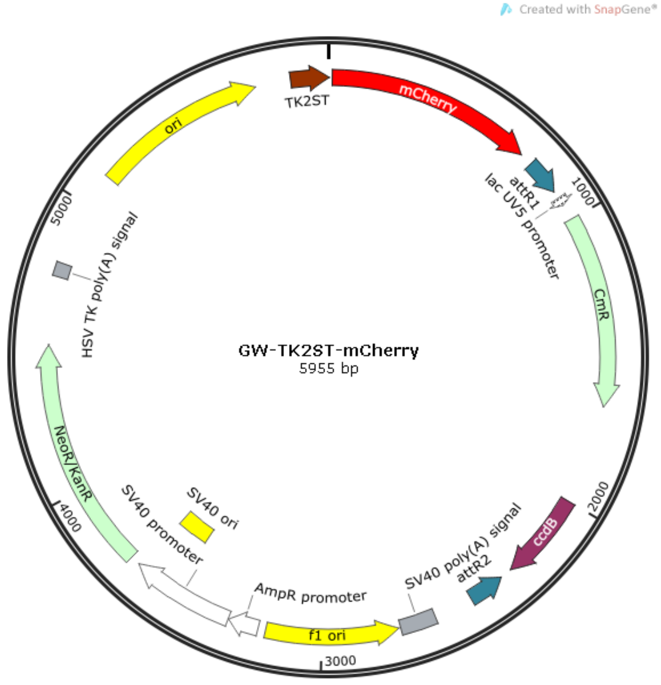
Created with SnapGene®
GW-TK2ST-mCherry
5955 bp
TK2ST
mCherry
attR1
lac UV5 promoter
CmR
ccdB
attR2
SV40 poly(A) signal
f1 ori
AmpR promoter
SV40 promoter
SV40 ori
NeoR/KanR
HSV TK poly(A) signal
ori
1000
2000
3000
4000
5000

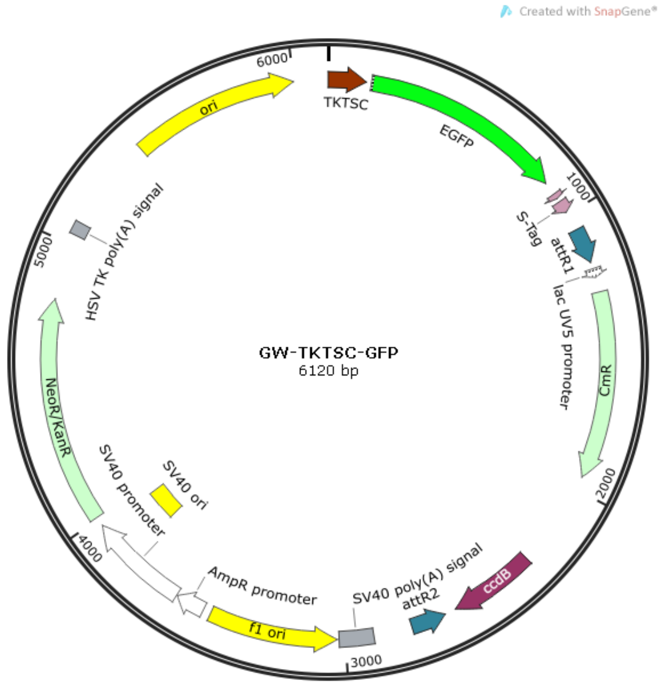
Created with SnapGene®
GW-TKTSC-GFP
6120 bp
TKTSC
EGFP
S-Tag
attR1
lac UV5 promoter
CmR
ccdB
attR2
SV40 poly(A) signal
f1 ori
AmpR promoter
SV40 promoter
SV40 ori
NeoR/KanR
HSV TK poly(A) signal
ori
1000
2000
3000
4000
5000
6000

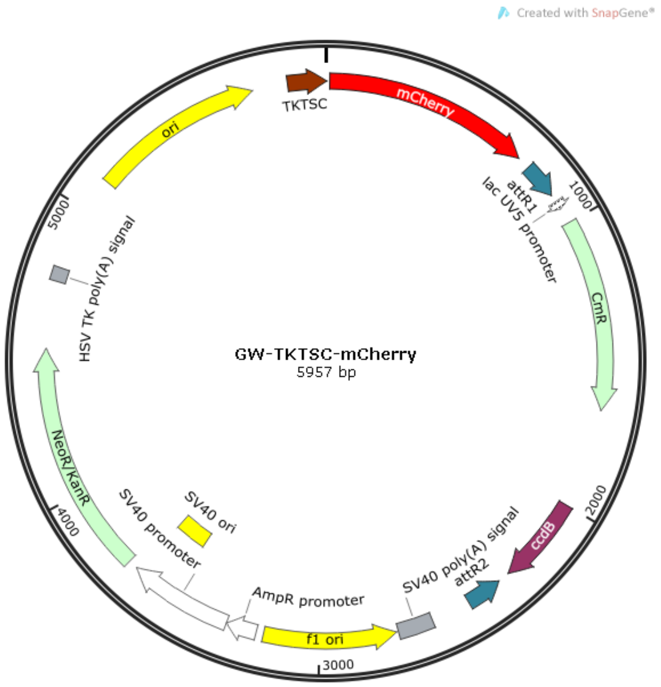
Created with SnapGene®
GW-TKTSC-mCherry
5957 bp
TKTSC
mCherry
attR1
lac UV5 promoter
CmR
ccdB
attR2
SV40 poly(A) signal
f1 ori
AmpR promoter
SV40 promoter
SV40 ori
NeoR/KanR
HSV TK poly(A) signal
ori
1000
2000
3000
4000
5000

Supplementary Table 1

Promoter Sequences

TK

TAATCGGTGGTTAGGGTTTGTCTGACGCGGGGGGAGGGGGAA
GGAACGAAACACTCTCATTCGGAGGCGGCTCGGGGTTTGGTCT
TGGTGGCCACGGGCACGCAGAAGAGCGCCGCGATCCTCTTAAG
CACCCCCCCGCCCTCCGTGGAGGCGGGGGTTTGGTCGGCGGGT
GGTAACTGGCGGGTCGCTGACTCGGGCGGGTCGCGCGCCCCAG
AGTGTGACCTTTTCGGTCTGCTCGCAGACCCCCGGGCGGCGCCG
CCGCGGCGGCGACGGGCTCGCTGGGTCCTAGGCTCCATGGGGA
CCGTATACGTGGACAGGCTCTGGAGCATCCGCACGACTGCGGT
GATATTACCGGAGACCTTCTGCGGGACGAGCCGGGTCACGCGG
CTGACGCGGAGCGTCCGTTGGGCGACAAACACCAGGACGGGG
CACAGGTACACTATCTTGTCACCCGGAGGCGCGAGGGACTGCA
GGAGCTTCAGGGAGTGGCGCAGCTGCTTCATCCCCGTGGCCCG
TTGCTCGCGTTTGCTGGCGGTGTCCCCGGAAGAAATATATTTGC
ATGTCTTTAGTTCTATGATGACACAAACCCCGCCCAGCGTCTTGT
CATTGGCGAATTCGAACACGCAGATGCAGTCGGGGCGGCGCG
GTCCCAGGTCCACTTCGCATATTAAGGTGACGCGTGTGGCCTCG
AACACCGAGCGACCCTGCAGCGACCCGCTTAGCTAG

TKΔSS

TAATCGGTGGTTAGGGTTTGTCTGACGCGGGGGGAGGGGGAA
GGAACGAAACACTCTCATTCGGAGGCGGCTCGGGGTTTGGTCT
TGGTGGCCACGGGCACGCAGAAGAGCGCCGCGATCCTCTTAAG
CACCCCCCCGCCCTCCGTGGAGGCGGGGGTTTGGTCGGCGGGT
GGTAACTGGCGGGTCGCTGACTCGGGCGGGTCGCGCGCCCCAG
AGTGTGACCTTTTCGGTCTGCTCGCAGACCCCCGGGCGGCGCCG
CCGCGGCGGCGACGGGCTCGCTGGGTCCTAGGCTCCATGGGGA
CCGTATACGTGGACAGGCTCTGGAGCATCCGCACGACTGCGGT
GATATTACCGGAGACCTTCTGCGGGACGAGCCGGGTCACGCGG
CTGACGCGGAGCGTCCGTTGGGCGACAAACACCAGGACGGGG
CACAGGTACACTATCTTGTCACCCGGAGGCGCGAGGGACTGCA
GGAGCTTCAGGGAGTGGCGCAGCTGCTTCATCCCCGTGGCCCG
TTGCTCGCGTTTGCTGGCGGTGTCCCCGGAAGAAATATATTTGC
ATGTCTTTAGTTCTATGATGACACAAACCCCGCCCAGCGTCTTGT
CATTGGCGAATTCGAACACGCAGATGCAGTCGGGGCGGCGCG
GTCCCAGGTCCACTTCGCATATTAAGGTGACGCGTGTGGCCTCG
AACACCGAGCTAG

Supplementary Table 2

Promoter Sequences

TK2ST

TAATACAAACCCCGCCCGAATTCGAACACGCAGATGCAGTCGG
GGCGGCGCGGTCCCAGGTCCACTTCGCATATTAAGGTGACGCG
TGTGGCCTCGAACACCGAGCGACCCTGCAGCGACCCGCTTAGCT
AG

TKTSC

TAATCGTCTTGTCATTGGCGAATTCGAACACGCAGATGCAGTCG
GGGCGGCGCGGTCCCAGGTCCACTTCGCATATTAAGGTGACGC
GTGTGGCCTCGAACACCGAGCGACCCTGCAGCGACCCGCTTAG
CTAG

TK2STΔSS

TAATACAAACCCCGCCCGAATTCGAACACGCAGATGCAGTCGG
GGCGGCGCGGTCCCAGGTCCACTTCGCATATTAAGGTGACGCG
TGTGGCCTCGAACACCGAGCTAG

TKTSCΔSS

TAATCGTCTTGTCATTGGCGAATTCGAACACGCAGATGCAGTCG
GGGCGGCGCGGTCCCAGGTCCACTTCGCATATTAAGGTGACGC
GTGTGGCCTCGAACACCGAGCTAG

TK*m*ΔSS

TAATGAACACGCAGATGCAGTCGGGGCGGCGCGGTCCCAGGTC
CACTTCGCATATTAAGGTGACGCGTGTGGCCTCGAACACCGAGC
TAG
